# Na^+^/H^+^-Exchanger Family as Novel Prognostic Biomarkers in Colorectal Cancer

**DOI:** 10.1155/2021/3241351

**Published:** 2021-11-01

**Authors:** Xin Zhou, Manman Jiang, Zhihong Liu, Mengmeng Xu, Nannan Chen, Ziyu Wu, Changji Gu, Eugene Chin, Xiaodong Yang

**Affiliations:** ^1^Department of General Surgery, The Second Affiliated Hospital of Soochow University, Suzhou, China; ^2^Institutes of Biology and Medical Science, Soochow University, Suzhou, China

## Abstract

**Background:**

The acidic characteristics of the tumor microenvironment (TME) are attributed to cancer cells' needs of metabolism which produce a large amount of H^+^. In order not to affect its own life activities, it needs to release H^+^ into the intercellular space through an efficient Na^+^/H^+^ exchanger. On account of the intestine whose physiological function is highly dependent on intestinal pH value, NHE family members may play a critical role in the occurrence and development of colorectal cancer (CRC).

**Methods:**

TCGA, GEPIA2, ONCOMINE, UALCAN, STRING, TIMER, Cytoscape, TargetScan, ENCORI, LncBase v.2, DNMIVD, HPA, and CellMiner^TM^ databases were used in our study.

**Results:**

The mRNA expressions of SLC9A1, SLC9A2, SLC9A3, and SLC9A9 were evidently lower in COAD than in normal samples; however, the mRNA expressions of SLC9A5, SLC9A8, and SLC9B2 were higher. Besides, mRNA expressions of NHE family were extremely associated with clinicopathological features, tumor immune microenvironment and stemness score, DNA methylation, and patient prognosis in COAD. Moreover, we conjectured that NHE family may play a role through MAPK or ErbB signaling pathway according to the results of GO/KEGG enrichment analysis. At last, we found that NHE family members were key factors of various kinds of cancers.

**Conclusion:**

Our study indicated that NHE family represented new diagnostic and therapeutic targets for CRC, which could have important significance for the clinical treatment of CRC.

## 1. Introduction

Aiming at cancer, a momentous problem in the history of human medicine, scientists around the world are constantly exploring new ways of treatment from various angles. In the early stage, people have done a lot of research on solid tumor itself, most of which are committed to inhibiting tumor proliferation and migration, improving autoimmunity, and drug treatment. However, with the deepening of research, we came to know the concept of tumor microenvironment (TME) [1]. This new field has been developed so far; it is no stranger to the typical states of hypoxia, acidity, high content of lactate, abundant blood vessels, and infiltrating immune cells in the local environment around the tumor. In addition, the characteristics of the acidic microenvironment have attracted even more researchers' curiosity. In order to sustain the high proliferation of cancer cells, anaerobic glycolysis and increased oxidative phosphorylation are selected for rapid energy supply which engenders a large amount of CO_2_ and H^+^ [2]. As a result, the pH value in the area near cancer cells is lower than that in normal tissues. Previous studies have shown that acidic cellular environment can lead to DNA strand breakage and histone deacetylation, thereby increasing genetic instability and increasing the probability of cell carcinogenesis [3, 4]. Moreover, the acidic environment also assists cancer cells in avoiding the attack of immune cells [5]. But this phenomenon does not signify that cancer cells are eosinophilic, and it is just that they can adapt to the acidic environment better than normal cells. For cancer cells, excessive H^+^ produced by metabolic preference in cancer cells will also restrain the enzymes of glucose metabolism, thus restricting the proliferation of itself [6]. However, its cunning lies in that cancer cells are equipped with a more efficient system to release H^+^ outside the cell and maintain the intracellular balance: Na^+^/H^+^-exchangers, Na^+^, HCO_3_^−^-cotransporters, and H^+^-ATPases [7, 8].

As an important transporter for maintaining cell acid-base equilibrium, Na^+^/H^+^ exchanger exists in all apparatuses of mammals [9]. Just like its name, it is responsible for absorbing extracellular Na^+^ in the form of active transport, discharging excess H^+^ produced by metabolism and other biochemical reactions, and maintaining the pH requirements of various biological reactions in the intracellular environment [10]. So far, 13 subtypes of Na^+^/H^+^ ion pumps have been found, belonging to a large family (NHE), named NHE1-5 (SLC9A1-5), NHE6-9 (SLC9A6-9), NHE10, NHE11 (SLC9C1, C2), NHA1, and NHA2 (SLC9B1, B2) [11, 12]. They are distributed in different regions of cells and engage in similar work. In addition to their common functions of intracellular alkalization and cell volume control, individual members of the NHE family have also been revealed to be closely related to the occurrence and development of common inflammatory diseases and even tumors [13]. NHE1, which is almost only located outside the plasma membrane, has been studied more. It has been found that it is anchored in the cytoskeleton to participate in cell migration and invasion [14]. Abnormal NHE1 mRNA levels have been detected in pathological samples of a variety of digestive system tumors [15]. Animal and cytological experiments have also proved that it does make outstanding contributions to life activities such as cell proliferation and apoptosis, but the explicit mechanism and clinical practice have not received conclusions and support to verify these findings [16]. In NHE family, except NHE3 and NHE7-9, there are few studies on other family members for reference. Among them, NHE3 has a small number of reports that it plays a role in enteric diseases [17]. NHE7 affects the development of pancreatic cancer [18] and breast cancer [19] by changing the acid-base balance of cells and the abnormal expression of NHE8 and NHE9 in colon cancer suggesting that it may have an important impact on the development and prognosis of cancer [20, 21]. In general, the physiological function of NHE family in cancer has yet to be explored.

As is known to all, the physiological functions of the nervous system and digestive system are extremely dependent on hydrogen ion exchange [22]. We found that there had been a great quantity of studies on the regulation of NHE family on the occurrence and development of glioma [23]. Nevertheless, there are few studies in colorectal cancer (CRC). CRC remains a global public hygiene problem as usual, leading to high cancer-related mortality. According to statistics, CRC accounts for about 10% of global cancer diagnosis and cancer-related deaths every year. Moreover, on the basis of the latest research statistics, CRC has become the second deadliest cancer in the world [24, 25]. Although surgery, chemotherapy, and radiotherapy are quite effective for patients in stages I and II, follow-up observation shows that the probability of 5-year survival has increased to 66%. Unfortunately, we all know that the insurmountable fatal point of CRC lies in the invasion and metastasis of cancer cells. However, there is no report that a targeted policy of screening and surveillance by colonoscopy will curb the rising incidence of colorectal cancer, and modern clinical medicine has failed to reach the level of limiting its metastasis [26]. The 5-year survival rate of patients with advanced colorectal cancer is only about 13% [27]. Nevertheless, many scholars have not stopped exploring strategies for the prevention and treatment of CRC. The treatment strategies have also made progress step by step from surgical resection to immunotherapy [27]. Even recently, a study pointed out that colorectal cancer can be prevented through the regulation of dietary strategies on microbiota remodulation [28]. Consequently, it is urgent to find new approaches to prevent and cure CRC.

Our study is inspired by the acidic characteristics of the tumor microenvironment, based on the NHE family that regulates the pH value inside and outside the cell, combined with the H + dependent physiological function of the intestine. Using the available data resources, we can comprehensively develop new ideas for the defense and treatment of colorectal cancer. At first, we used the data from TCGA to analyze the mRNA expression of 10 subtypes of NHE family in COAD relative to the adjacent cancer, as well as the clinicopathological related effects (since SLC9B1, SLC9C1, and C2 are only expressed in male testis, they are not within the scope of this study). Immediately, we found 90 proteins interacting with NHE family members through STRING and speculated that NHE family members may participate in the reaction mechanism. Certainly, whether NHE family members affect the number and function of infiltrated immune cells and stem cells in the tumor microenvironment, we also made a full analysis using TCGA and TIMER databases. Then we utilized Cytoscape software to structure the ceRNA network of NHE family members. The screened ceRNA may become a new method for cancer treatment. In addition, we found the correlation between DNA methylation in COAD and NHE family members through DNMIVD and obtained IHC images of NHE family members in COAD and normal colon tissues in HPA. The cure and survival rate are the most direct criteria for the research and treatment of any disease. Therefore, we made a survival analysis related to COAD for NHE family. In addition to COAD, we also used R-package “ggpubr” to understand the expression of NHE family in other cancers. The drug sensitivity of NHE family is a test for its future clinical application, so we studied the data obtained from CellMiner^TM^ with “limma,” “ggplot2,” and “ggpubr.” Finally, all our results and graphs were completed by R software (version 4.0.5), and the *p* value of *t*-test < 0.05 was considered statistically significant.

## 2. Materials and Methods

### 2.1. Differentially Expressed NHE Family in COAD

All data of the gene expression RNA-seq and stemness score (RNA based) of 33 cancers were from the database UCSC Xena (https://xena.ucsc.edu/) [29]. We used heatmap and grid map to show the expression in 18 kinds of cancer and correlation of NHE family members. R-packages used in this study were “pheatmap” and “corrplot.”

GEPIA2 (https://gepia2.cancer-pku.cn/) is a powerful database platform which can visualize all kinds of data of 9736 tumors and 8587 normal samples from the TCGA and the GTEx projects [30]. In this study, we searched ten NHE family members in “Multiple Genes Comparison” which was in “Expression DIY.”

ONCOMINE database (https://www.oncomine.org/) is an online database containing a huge amount of the most comprehensive gene microarray data [31]. A great quantity of gene expression data of NHE family members in COAD tissues and normal colon tissues was obtained from ONCOMINE database.

### 2.2. Clinicopathological Analysis of NHE Family in COAD

UALCAN (https://ualcan.path.uab.edu/) is a comprehensive platform for deep mining and visualization of TCGA data [32]. Users can easily obtain comprehensive clinicopathological analysis data of gene from UALCAN. Therefore, we used “TCGA” of UALCAN to analyze the correlation between the mRNA expression of NHE family members in COAD and individual cancer stages and nodal metastasis status.

### 2.3. Construction of the PPI Network

STRING (https://string-db.org/) is a database that clearly displays known and predicted protein-protein interactions [33]. At present, STRING 11.0 contains 24,584,628 proteins from 5,090 organisms. In our study, we made use of STRING to construct a PPI network of NHE family members and the combination score >0.4 was considered credible.

### 2.4. GO/KEGG Enrichment Analysis

GO/KEGG enrichment analysis is universally deemed the most reliable method for gene function annotation. In order to find out what biological processes NHE family members are involved in, we used “clusterProfiler” package in R to carry out GO/KEGG enrichment analysis of NHE family members and proteins interacting with them (90 genes). GO functional enrichment analysis consisted of three parts: biological processes (BP), cellular components (CC), and molecular functions (MF). KEGG enrichment analysis was chiefly used to conjecture those pathways involved in NHE family members.

### 2.5. Relevance Analysis of NHE Family Members Expression with TME and Stromal Score

In recent studies, whether gene expression affects the tumor microenvironment (TME) has become a reliable criterion to evaluate the importance of a gene. We used “estimate” and “limma” packages in R to analyze NHE family members expression, which could calculate the relationship between NHEs and stromal/immune cells in TME. After that, “cor.Test” command and R-package “limma” were used to assess the connection between NHEs and RNA stemness score (RNAss) and DNA stemness score (DNAss). All these analyses were visualized by R-packages “corrplot,” “reshape2,” “ggpubr,” and “ggplot2.”

### 2.6. Construction of ceRNA Network

Pandolfi P.P and his team proposed a hypothesis “ceRNA Network” in 2011 [34]. CeRNA Network refers to the large-scale regulatory network formed between coding RNA and noncoding RNA. This hypothesis enriches our cognition of diseases and puts forward new ideas for research. Based on the successful experience of Yang F.B [35], we inversely predicted the ceRNA network of NHE family in our study. TargetScan (https://www.targetscan.org/) is a powerful miRNA prediction tool containing multiple species information [36]. LncBase v.2 (https://carolina.imis.athena-innovation.gr/diana_tools/web/) is a special tool developed by DIANA LAB to predict the regulatory relationship between miRNA and lncRNA. ENCORI (https://starbase.sysu.edu.cn/) is a superb open-source platform which identifies more than 1.1 million miRNA-ncRNA, 2.5 million miRNA-mRNA, 2.1 million RBP-RNA, and 1.5 million RNA-RNA interactions from multidimensional sequencing data [37]. At first, we successfully predicted all the miRNAs of NHE family using TargetScan. Then, we intersected the miRNAs of each NHE family member (number of repetitions ≥ 7). Next, we used LncBase v.2 to predict the lncRNA of these miRNAs (Pr.score>0.6). Meanwhile, we searched the expression and survival analysis of these miRNAs in COAD on ENCORI. At last, ceRNA network was constructed by Cytoscape software.

### 2.7. Immune Infiltration Analysis of NHE Family

In recent years, tumor regulation by immune cells has become a major topic of discussion. TIMER (https://cistrome.shinyapps.io/timer/) is a synthetic database containing vast quantities of information about immune cell infiltration in various tumors [38]. In our study, we estimated the relevance between NHE family and the infiltration of 6 kinds of immune cells (B cells, CD4+ T cells, CD8+ T cells, neutrophils, macrophages, and dendritic cells) in COAD.

### 2.8. Methylation Analysis of NHE Family

DNMIVD (https://www.unimd.org/dnmivd/) is a user-friendly interactive visualization database for DNA methylation [39]. In this study, it was used to obtain the relevance between DNA methylation and NHE family members in COAD. Student's *t*-test adjusted *p* value ≤0.05 and |beta difference|>0.2.

### 2.9. Immunohistochemistry (IHC) of NHE Family in COAD

The Human Protein Atlas (https://www.proteinatlas.org) is the largest protein information database at present, which provides great assistance for the study of researchers all over the world [40]. This database currently covers 17165 proteins and nearly 20 tumors. In our study, IHC images of NHE family members in COAD tissues and normal colon tissues were obtained from HPA.

### 2.10. Survival Analysis

Survival analysis is the most clinically significant data for gene research. Overall survival (OS) line charts of NHE family members in COAD were obtained from GEPIA2. The clinical data included in this analysis were 9736 tumors and 8587 normal samples from the TCGA and the GTEx projects.

### 2.11. Drug Sensitivity of NHE Family

The chemosensitivity per patient has always been a critical factor restricting the efficacy of CRC chemotherapy. In our study, we downloaded drug sensitivity processed data from the CellMiner^TM^ database (https://discover.nci.nih.gov/cellminer/home.do) [41]. CellMiner™ database covers drug sensitivity information for 60 cancer cell lines. All data were analyzed and visualized with R-packages “impute,” “limma,” “ggplot2,” and “ggpubr.”

### 2.12. Pan-Cancer Analysis

We showed the expression data of NHE family members in 18 cancers and adjacent tissues from TCGA database by box diagram. All gene expression data were downloaded from UCSC Xena. Then we used R-package “ggpubr” to get all the charts we need.

### 2.13. Statistical Analysis

In our study, all plots were completed by R software (version.4.0.5). *P* value < 0.05 of *t*-test was considered statistically significant.

## 3. Results

### 3.1. Deviant Expression of NHE Family in COAD

**Figure 1:**
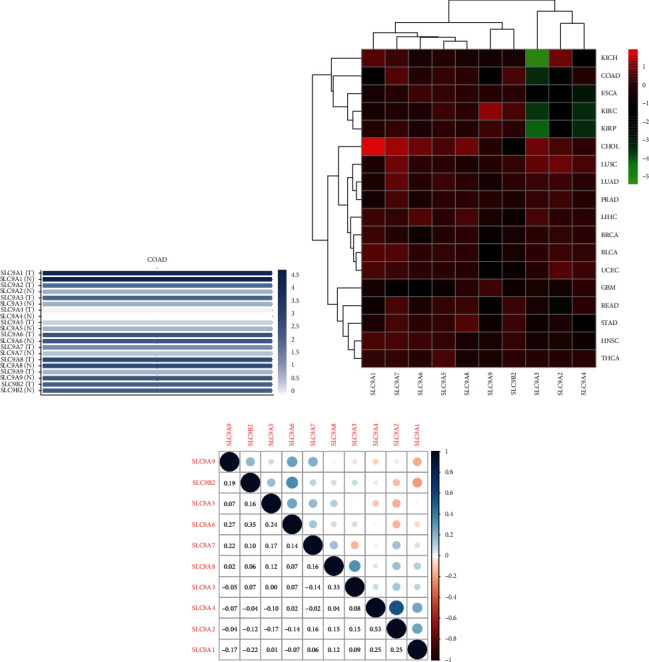
NHE family expression and correlation in COAD. (a) The expression of NHE family members in COAD and normal samples from GEPIA2. (b) NHE family expression in different cancers from TCGA. Red and green indicate high and low expression, respectively. (c) Interaction of NHE family members. Blue dots mean negative correlation, and red dots mean positive correlation.

The difference of gene transcription level is the most basic standard to evaluate the significance of genes. By analyzing the data from multiple databases, we found that there were conspicuous differences in the expression of NHE family members in CRC tissues and normal intestinal tissues. The bar plot generated by GEPIA2 clearly showed that the expressions of SLC9A2, SLC9A3, and SLC9A9 in COAD tissues were obviously lower than those in normal tissues, but SLC9A5 and SLC9A7 in cancer were distinctly higher than those in normal tissues (Figure 1(a)). Then the heatmap made of TCGA data further validates our former findings (Figure 1(b)). Furthermore, it was rather remarkable that the difference of SLC9A3′ expression in COAD was extremely obvious, which could be worthy of our intensive study in the future. Next, we found many datasets in ONCOMINE, the database with the most abundant datasets, which showed that the expressions of NHE family members were significantly different between colorectal cancer and normal tissues (Table 1). Finally, we analyzed the correlation between NHE family members. The outcome showed that SLC9A2 and SLC9A4, SLC9A3 and SLC9A8, and SLC9A6 and SLC9B2 had striking positive correlation, and SLC9A1 was negatively correlated with SLC9B2 (Figure 1(c)).

### 3.2. Effect of NHE Family on Invasion and Metastasis of COAD

**Figure 2:**
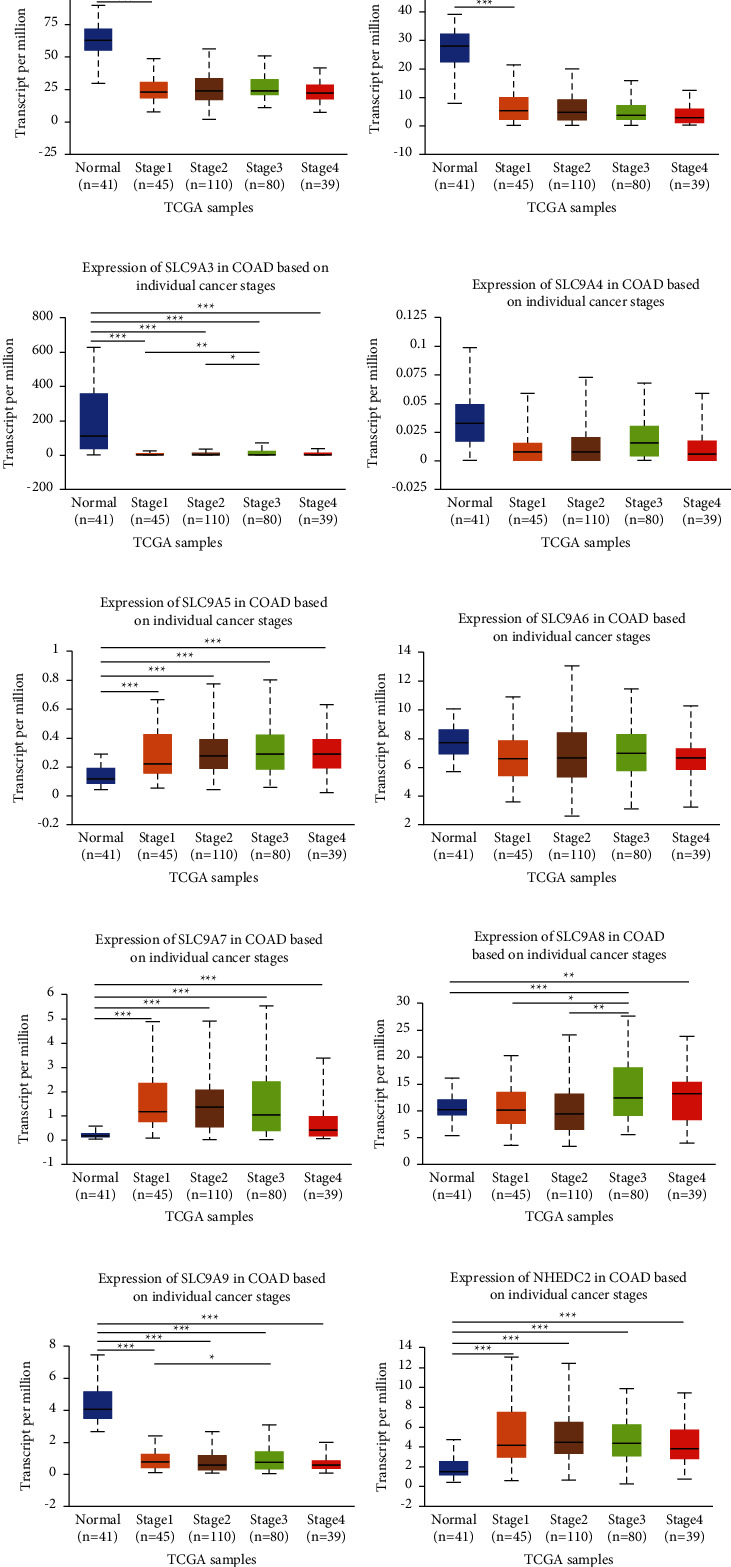
Correlation analysis of NHE family with tumor stage in COAD. (a–j) The horizontal axis represents tumor stage, and the longitudinal axis represents the expression of mRNA of NHE family. The symbols “^*∗*^,” “^∗∗^,” and “^∗∗∗^” indicate *p* values of <0.05, <0.01, and <0.001, respectively. Expression of (a) SLC9A1, (b) SLC9A2, (c) SLC9A3, (d) SLC9A4, (e) SLC9A5, (f) SLC9A6, (g) SLC9A7, (h) SLC9A8, (i) SLC9A9, and (j) NHEDC2 in COAD based on individual cancer stages.

**Figure 3:**
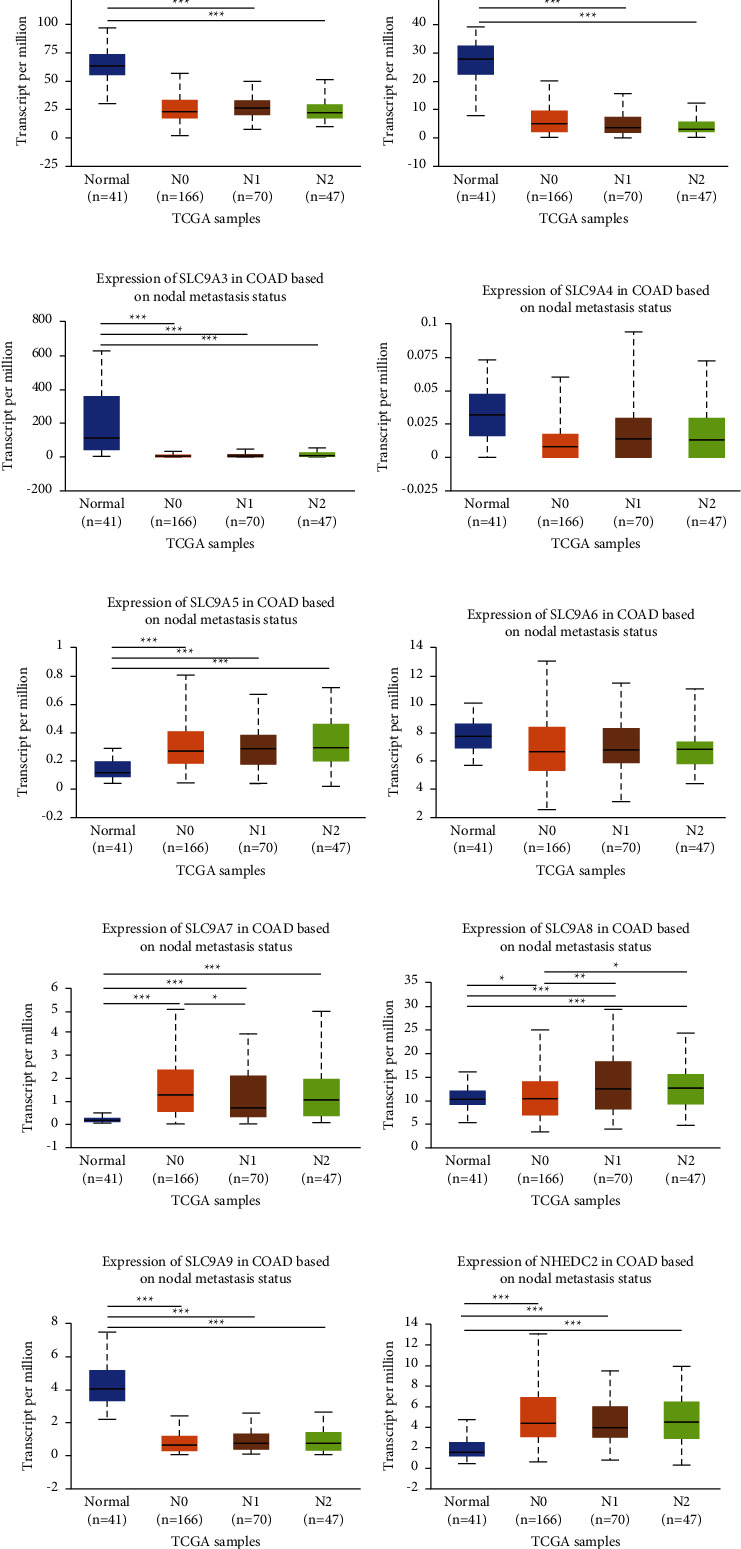
Correlation analysis of NHE family with nodal metastasis status in COAD. (a–j) The horizontal axis represents nodal metastasis status, and the longitudinal axis represents the expression of mRNA of NHE family. The symbols “^*∗*^,” “^∗∗^,” and “^∗∗∗^” indicate *p* values of <0.05, <0.01, and <0.001, respectively. Expression of (a) SLC9A1, (b) SLC9A2, (c) SLC9A3, (d) SLC9A4, (e) SLC9A5, (f) SLC9A6, (g) SLC9A7, (h) SLC9A8, (i) SLC9A9, and (j) NHEDC2 in COAD based on nodal metastasis status.

As we all know, individual tumor stage and lymph node metastasis could intuitively display the condition of tumor invasion and metastasis. The outcome of UALCAN showed that, except SLC9A4 and SLC9A6, the mRNA expression of other members prominently affected tumor stage (Figure 2) and lymph node metastasis (Figure 3). Furthermore, these effects were more obvious in advanced patients. All in all, the above results indicated that NHE family may be the critical factor affecting the invasion and metastasis of CRC.

### 3.3. PPI Network Construction and GO/KEGG Enrichment Analysis of NHE Family

**Figure 4:**
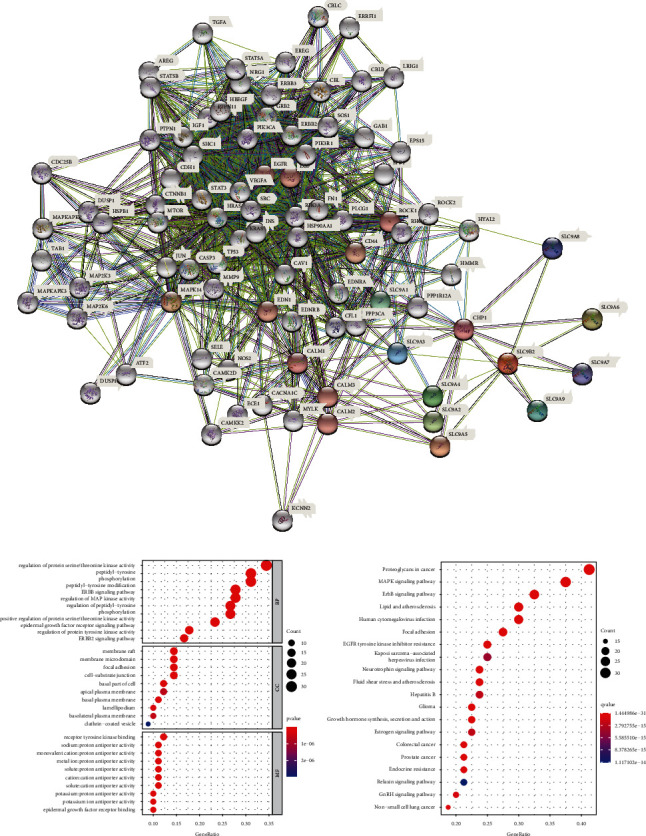
PPI network and GO/KEGG enrichment analysis of NHE family. (a) PPI network of NHE family constructed by STRING including 90 genes. (b) GO functional enrichment analysis (BP, CC, and MF). (c) KEGG pathway enrichment analysis.

Construction PPI network could help us better understand the interaction between NHE family and other proteins. Using STRING database, we got a complex PPI network containing 90 proteins (Figure 4(a)).

In order to better understand the function of NHE family, we performed GO/KEGG enrichment analysis on 90 genes obtained from PPI network (Figures 4(b) and 4(c)). GO enrichment analysis results showed that the biological processes (BP) involved by NHE family mainly included GO:0071900 (regulation of protein serine/threonine kinase activity), GO:0018212 (peptidyl-tyrosine modification), GO:0038127 (ErbB signaling pathway), and GO:0043405 (regulation of MAP kinase activity). Cellular components (CC) analysis indicated that NHE family were mainly related to GO:0045121 (membrane raft), GO:0098857 (membrane microdomain), GO:0005925 (focal adhesion), and GO:0030055 (cell-substrate junction). Molecular functions (MF), including GO:0030971 (receptor tyrosine kinase binding), GO:0015385 (sodium: proton antiporter activity), GO:0005451 (monovalent cation: proton antiporter activity), and GO:0051139 (metal ion: proton antiporter activity), were prominently related to the NHE family alterations. Besides, NHE family were extremely likely to participate in hsa04012 (ErbB signaling pathway), hsa05205 (proteoglycans in cancer), hsa04010 (MAPK signaling pathway), hsa05417 (lipid and atherosclerosis), and other pathways. Significantly, KEGG pathway enrichment analysis showed that NHE family were highly associated with hsa05210 (colorectal cancer), which also corroborated our guess. All GO/KEGG analysis results are listed in Table 2.

### 3.4. Relevance between mRNA Expression of NHE Family and TME and Stromal Score in COAD

**Figure 5:**
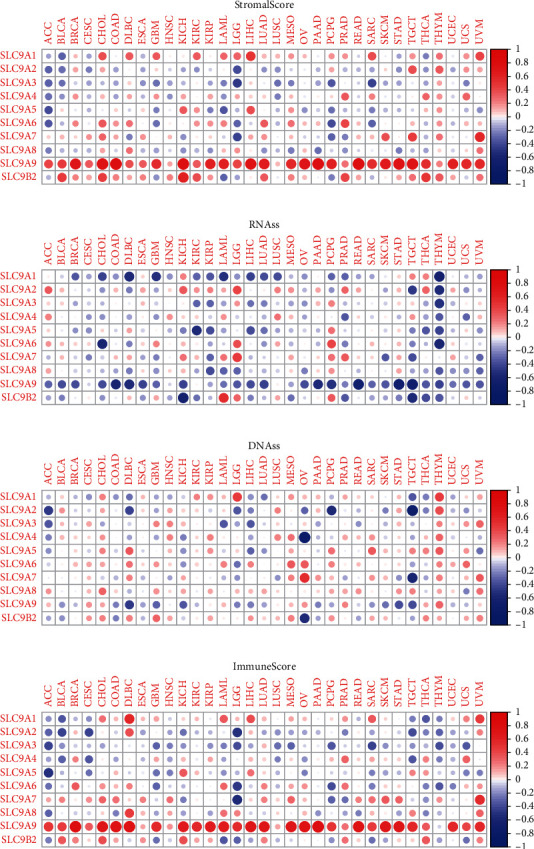
Correlation analysis of NHE family with TME and stromal score. NHE family members related to stromal score (a), RNAss (b), DNAss (c), and immune score (d). Blue dots mean negative correlation, and red dots mean positive correlation.

In recent years, researchers have increasingly been conscious of the importance of TME for tumor development. As an ion transporter, NHE family must be closely related to TME. Based on the dot-matrix plot obtained from the evaluation of NHE family, we found that most NHE family members were positively correlated with stromal score (Figure 5(a)) and immune score (Figure 5(d)) in COAD. Inside, the correlation degree of SLC9A9 was the most prominent, while SLC9A8 was significantly negatively correlated with immune score, which were valuable research subjects. On the contrary, the overwhelming majority of NHE family had a negative relevance to RNAss (Figure 5(b)) and DNAss (Figure 5(c)) in COAD.

### 3.5. hsa-miR-149-3p and hsa-miR-5193 May Be Pivotal miRNAs in ceRNA Network

**Figure 6:**
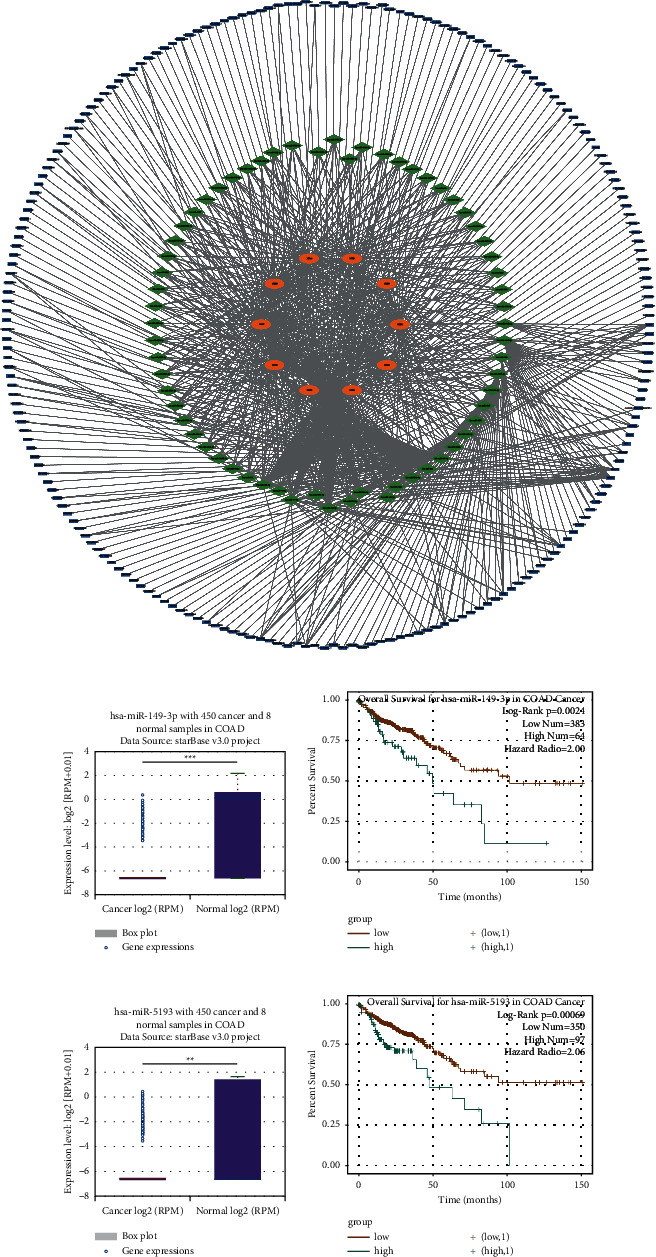
The ceRNA network of NHE family and two critical miRNAs found by the ENCORI database. (a) The ceRNA network of NHE family was constructed by Cytoscape. Orange oval represents mRNA, green diamond represents miRNA, and blue rectangle represents LncRNA. (b) The expression of hsa-miR-149-3p in COAD and normal samples from the ENCORI database. (c) The result of overall survival for hsa-miR-149-3p in COAD from the ENCORI database. (d) The expression of hsa-miR-5193 in COAD and normal samples from the ENCORI database. (e) The result of overall survival for hsa-miR-5193 in COAD from the ENCORI database.

Based on the prediction ability of TargetScan, we inversely predicted the miRNA of NHE family. After the intersection of these miRNAs, 65 miRNAs were obtained (number of repetitions ≥ 7). Then 215 LncRNA associated with these miRNAs were predicted through LncBase v.2. After that, we used Cytoscape to build a ceRNA network of NHE family (Figure 6(a)). After searching these 65 miRNAs on ENCORI, we found that only has-miR-149-3p and has-miR-5193 could reduce the survival rate of patients and their differential expression in cancer and normal sample was notable (Figures 6(b)–6(e)).

### 3.6. NHE Family Regulated the Infiltration of Immune Cells in COAD

**Figure 7:**
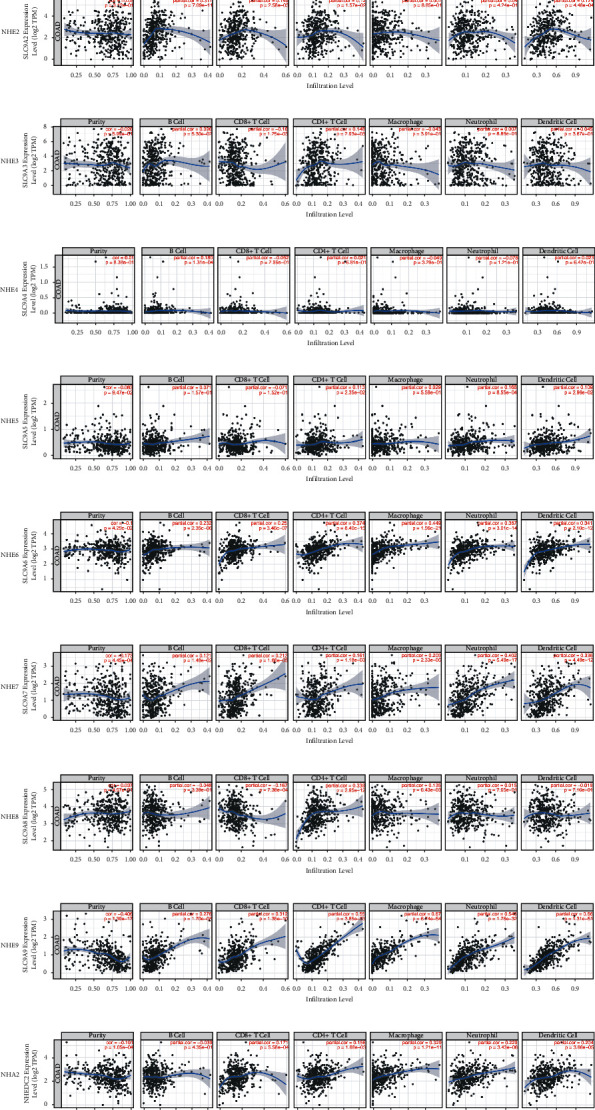
Relationship between NHE family expression and the infiltration of immune cells in COAD from the TIMER database.

The results of TIMER database showed that SLC9A1, SLC9A2, SLC9A6, SLC9A7, SLC9A9, and SLC9B2 were associated with six kinds of immune cells (B cells, CD4+ T cells, CD8+ T cells, neutrophils, macrophages, and dendritic cells) infiltrating COAD (Figure 7). Inside, SLC9A6 and SLC9A9 were the most prominent. Furthermore, except for SLC9A4, NHE family were closely related to CD4+ T cells. Therefore, CD4+ T cells were most likely the key factor for NHE family to regulate tumor development in COAD.

### 3.7. NHE Family Methylations Were Strongly Related to COAD

**Figure 8:**
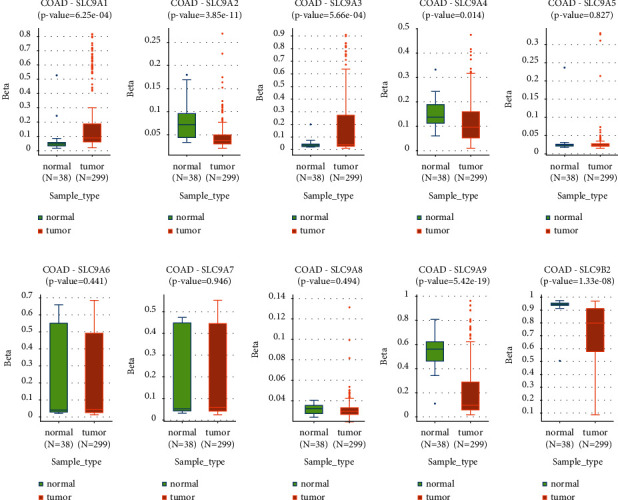
Change in NHE family methylation in COAD and normal tissues from the DNMIVD database.

DNA methylation is one of the key factors in tumorigenesis and development. The outcomes of DNMIVD database displayed that SLC9A1 and SLC9A3 showed dramatically higher methylation levels in COAD compared with normal tissues (Figures 8(a) and 8(c)); however, SLC9A2, SLC9A4, SLC9A9, and SLC9B2 represented lower methylation levels in COAD (Figures 8(b), 8(d), 8(i), and 8(j)).

### 3.8. Different Expressions of NHE Family Proteins in COAD Patients

**Figure 9:**
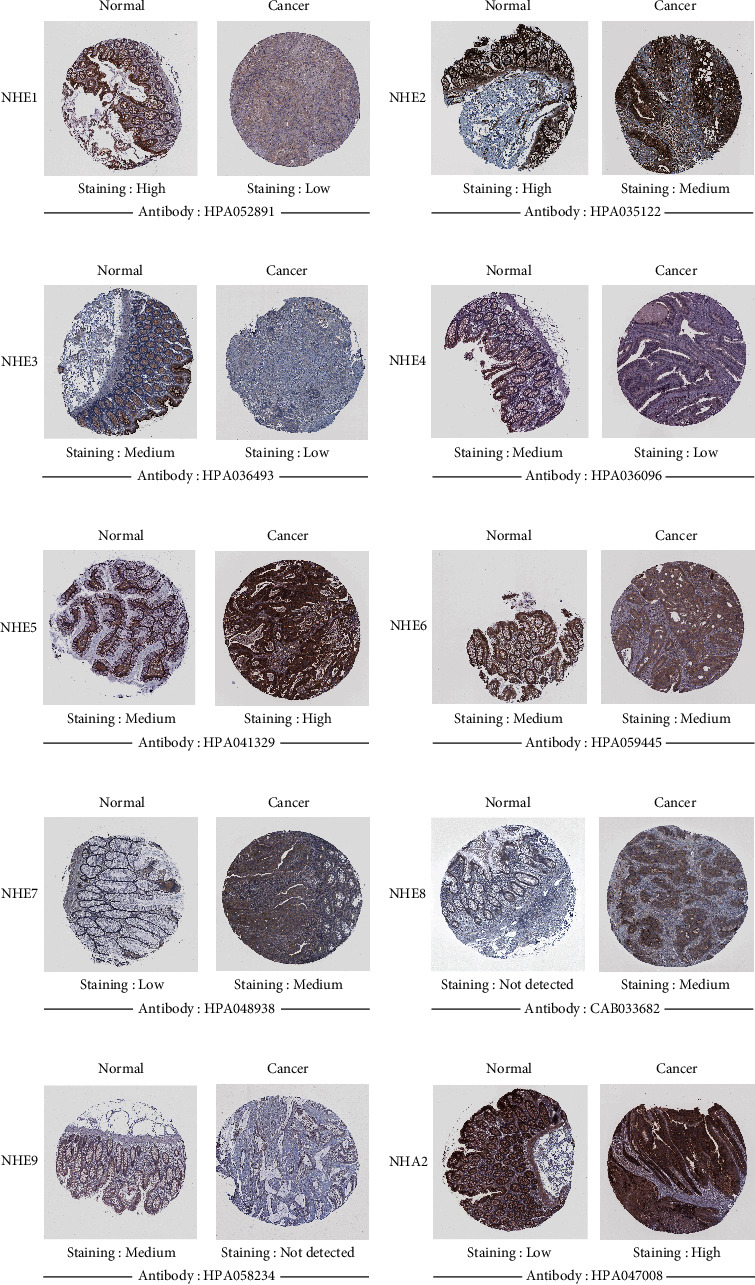
The expression of NHE family proteins in COAD from HPA (IHC).

The change of protein expression in clinical samples is the basis of clinical significance. The IHC plots of NHE family got from HPA database showed that SLC9A1, SLC9A2, SLC9A3, SLC9A4, and SLC9A9 were lower expressed in COAD than in normal colon, and the proteins of SLC9A5, SLC9A7, SLC9A8, and SLC9B2 were significantly upregulated in COAD (Figure 9).

### 3.9. Different Expression Levels of NHE Family Affected the Survival Rate of COAD Patients

**Figure 10:**
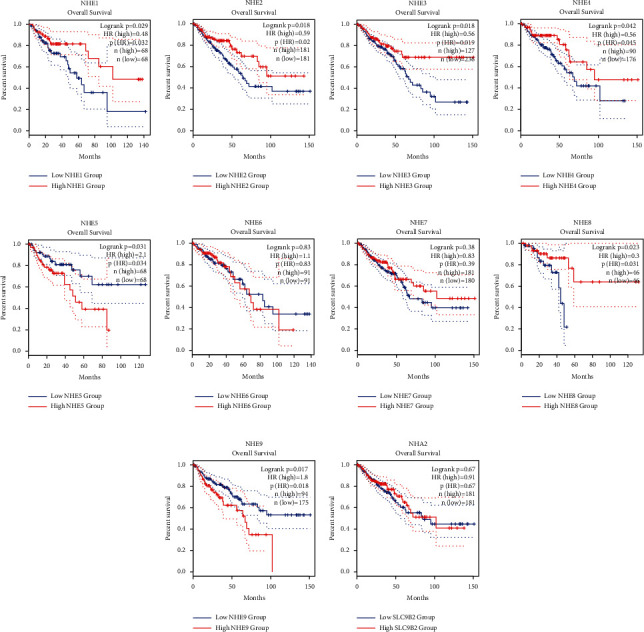
The prognostic value of mRNA expression of NHE family in COAD from GEPIA2. (a) NHE1. (b) NHE2. (c) NHE3. (d) NHE4. (e) NHE5. (f) NHE6 (g) NHE7. (h) NHE8. (i) NHE9. (j) NHA2.

Improving the prognosis of patients is one of the most important criteria to evaluate the significance of a gene. As we expected, the results of GEPIA2 showed that most of NHE family members were related to the prognosis of COAD patients (Figure 10). *p* values of SLC9A1, SLC9A2, SLC9A3, SLC9A4, SLC9A5, SLC9A8, and SLC9A9 were 0.032, 0.02, 0.019, 0.045, 0.034, 0.031, and 0.018, respectively. Among them, the high expression of SLC9A5 and SLC9A9 decreased the survival rate of COAD patients.

### 3.10. NHE Family Affected Multiple Drug Sensitivity

**Figure 11:**
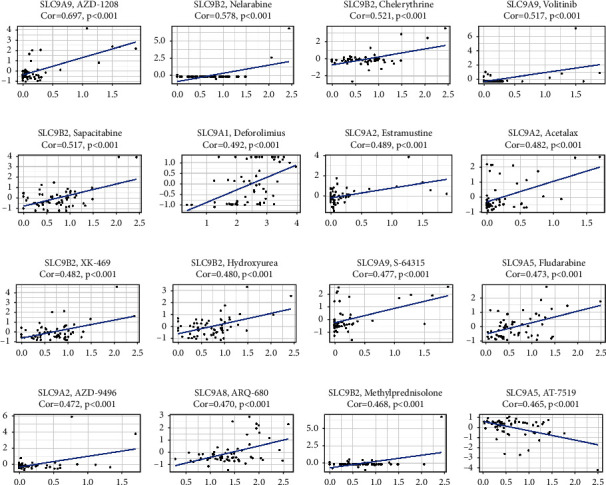
Drug sensitivity analysis of NHE family from the CellMiner^TM^ database.

The correlation analysis of drug sensitivity indicated that SLC9A1 was positively associated with Deforolimius sensitivity (Cor = 0.492); SLC9A2 was positively associated with Acetalax (Cor = 0.482) and AZD-9496 (Cor = 0.472); SLC9A5 was positively associated with Fludarabine (Cor = 0.473) and negatively associated with AT-7519 (Cor = -0.465); SLC9A8 was positively associated with ARQ-680 (Cor = 0.470); SLC9A9 was positively associated with AZD-1208 (Cor = 0.697), Volitinib (Cor = 0.517), Estramustine (Cor = 0.489), and S-64315 (Cor = 0.477); SLC9B2 was positively associated with Nelarabine (Cor = 0.578), Chelerythrine (Cor = 0.521), Sapacitabine (Cor = 0.517), XK-469 (Cor = 0.482), Hydroxyurea (Cor = 0.480), and Methylprednisolone (Cor = 0.468) (Figure 11). *p* value < 0.001 was considered significant.

### 3.11. NHE Family Were Key Factors in a Variety of Tumors

**Figure 12:**
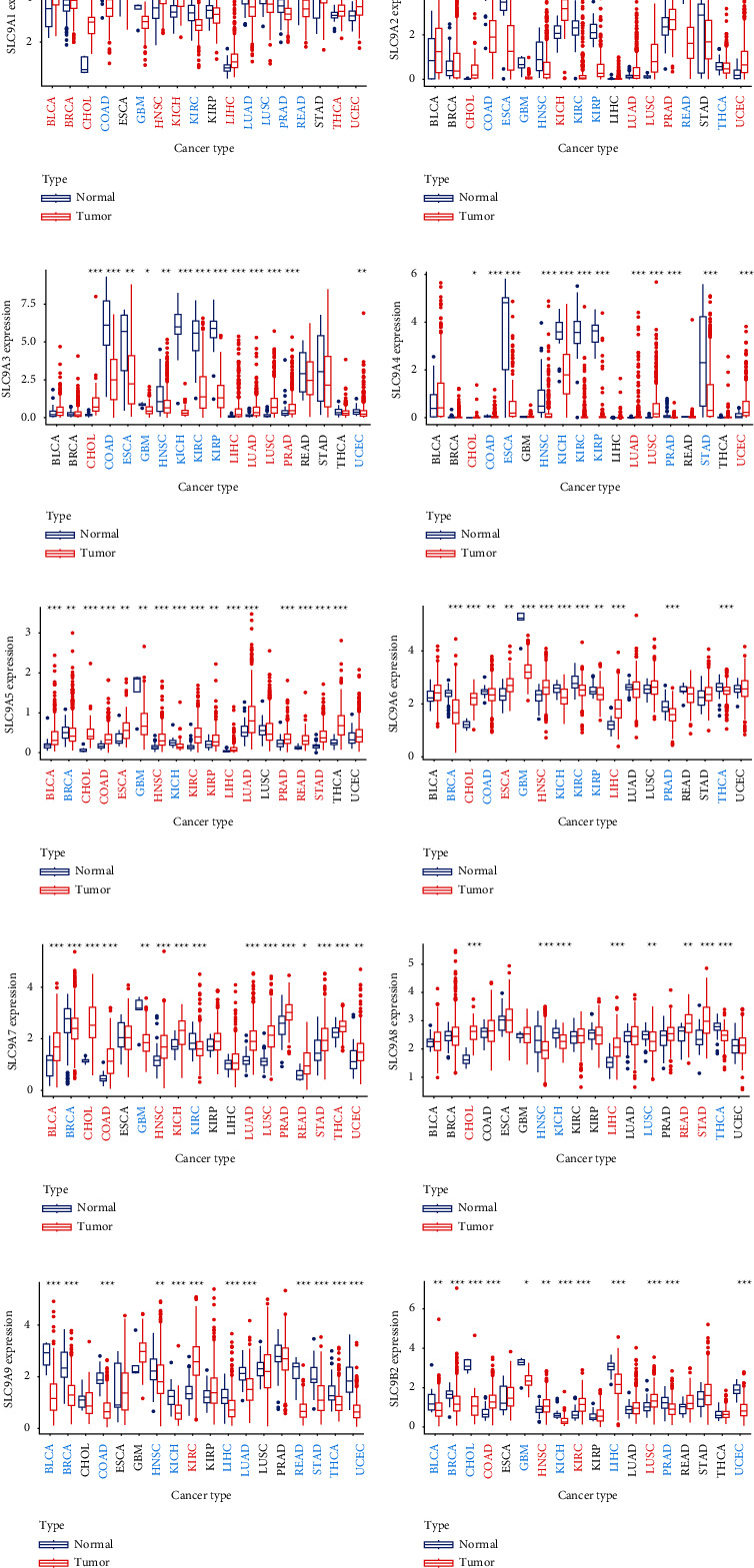
Pan-cancer analysis of NHE family from TCGA data. Red cancer names mean high expression, and blue cancer names mean low expression. The symbols “^*∗*^,” “^∗∗^,” and “^∗∗∗^” indicate *p* values of <0.05, <0.01, and <0.001, respectively.

The outcome of pan-cancer analysis showed that the expression of NHE family members in various tumors was significantly different from that in normal tissues (Figure 12). “^*∗*^,” “^∗∗^,” and “^∗∗∗^” indicated *p* values <0.05, <0.01, and <0.001, respectively.

## 4. Discussion

It has been well known that colorectal cancer may develop in patients with distinct intestinal diseases such as Inflammatory Bowel Diseases, Microscopic Colitis, and Irritable Bowel Syndrome [42]. All these intestinal diseases are closely related to changes in the microenvironment, such as oxygen metabolism, adenosine metabolism, and the synthesis and breakdown of inflammatory factors [43]. Therefore, alterations in the microenvironment are likely to lead to the progression of different intestinal diseases to colorectal cancer. Based on this speculation, the concept of TME emerges as the times require. Since the concept of TME was formally put forward in the 1980s, TME has been widely recognized as the most critical factor affecting the occurrence and development of tumors. TME plays a regulatory role in tumor development in many ways, such as nutrient and metabolic waste transport, immune escape, and pH change. The acidic tumor microenvironment acts as the driver of tumor for the following four reasons. At first, acid could destroy double-stranded DNA, resulting in gene mutation, which promotes the occurrence of tumor. Secondly, cancer cells are more adaptable to the acidic microenvironment than normal cells. Next, the acidic microenvironment will shorten the cell cycle to make cell proliferation abnormal. Lastly, the acidic microenvironment reduces the adhesion between cells, which could promote cancer metastasis. SLC9 family genes encode Na^+^/H^+^ exchangers proteins which play a significant role in regulating pH of intracellular and extracellular environment. As we all know, the physiological mechanisms of the human nervous system and digestive system are closely related to H^+^ exchange. At present, there have been a large number of studies on the regulation of glioma by NHE family members, but, interestingly, the role of NHE family in CRC is rarely mentioned. Therefore, it is a worthy study to analyze mRNA expression, prognostic roles, and correlation of NHE family in CRC.

In our study, we used TCGA data, GEPIA2, and ONCOMINE to compare the expressions of mRNA of NHE family in COAD and normal tissues. The results from TCGA and GEPIA2 showed that the mRNA expressions of SLC9A1, SLC9A2, SLC9A3, and SLC9A9 were evidently lower in COAD compared to normal samples; however, the mRNA expressions of SLC9A5, SLC9A8, and SLC9B2 were higher. At the same time, the data from ONCOMINE, a powerful database, validated our findings again. In addition, we found that there were many datasets showing the difference of SLC9A1, SLC9A2, SLC9A9, and SLC9B2. Furthermore, we not only observed this phenomenon at the mRNA level but also verified it at the protein level. The IHC of NHE family from HPA showed that the protein expressions of NHE1, NHE2, NHE3, NHE4, and NHE9 were significantly lower in COAD samples than in normal samples, and the results of NHE5, NHE7, NHE8, and NHA2 were just the opposite. The differences in mRNA and protein levels were only the basis of our study and whether there was clinical significance could better reflect the importance of the research. The results of UALCAN database showed that mRNA expressions of most NHE family members could affect tumor stage and lymph node metastasis. In addition, survival analysis of NHE family from GEPIA2 showed that high expressions of SLC9A1, SLC9A2, SLC9A3, SLC9A4, and SLC9A8 could improve the prognosis of COAD patients and low expressions of SLC9A5 and SLC9A9 could improve the prognosis of COAD patients. Drug sensitivity is another important aspect of tumor research. The results of our study showed that NHE family members could improve the sensitivity of various chemotherapeutic drugs. All the above results showed that NHE family were important regulatory genes in COAD.

Since we had understood the importance of NHE family in COAD, it was imperative to study the mechanism and physiological characteristics of NHE family. Construction of PPI network provided us 80 genes that interacted with NHE family members. Then, we used these 80 genes for GO/KEGG enrichment analysis and obtained four bubble plots. According to the results of KEGG pathway enrichment analysis, we conjectured that NHE family may play a role through MAPK or ErbB signaling pathway. Furthermore, GO enrichment analysis showed that NHE family members took part in physiological processes such as regulation of protein serine/threonine kinase activity, peptidyl-tyrosine phosphorylation, peptidyl-tyrosine modification, regulation of MAP kinase activity, protein tyrosine kinase binding, kinase regulator activity, and protein serine/threonine kinase activity. However, the specific regulation mechanism of NHE family in COAD still needs our follow-up research.

In recent years, TME, DNA methylation, and immune cell infiltration have become important directions in tumor research. Interestingly, in our study, we found that NHE family was significantly associated with TME and immune cell infiltration in COAD. What is more, the effect of mRNA expressions of NHE family on tumor immunity was extremely enormous. Consequently, it was very likely that NHE family regulated the development of COAD through immune escape. Among the six kinds of immune cells, CD4+ T cells were the most closely related to NHE family. Therefore, CD4+ T cells would be a major research direction of NHE family in tumor immunity. In addition, the methylation levels of NHE family members had also changed dramatically in COAD, which could provide us a new idea of NHE family research.

MicroRNA (miRNA) are small, noncoding RNAs of approximately 21–24 nucleotides in length. Thanks to the rapid development of microarray and RNA sequencing (RNA-seq) technology, more and more miRNAs are being uncovered to play an important role in the development of tumors [44]. Naturally, there are countless studies on miRNA regulation of colorectal cancer [45, 46]. At the same time, it has been shown that gene expression and function of NHE family members are regulated by miRNAs [47, 48]. Consequently, we inversely constructed the ceRNA network of NHE family in COAD and found two critical miRNAs, which could provide a basis for future research.

At last, the outcome of pan-cancer analysis showed that the expressions of NHE family members in cancer tissues were extremely different from those in normal tissues, which indicated that NHE family members may play a great role in a variety of tumors.

Obviously, there were still many deficiencies in our study. On the one hand, we only used online databases and datasets for all the analysis, so there was a lack of experimental data to verify our findings. In this regard, we will continue to conduct some in vivo and in vitro experiments. On the other hand, we did not find the specific mechanism of NHE family in regulating CRC. The mechanism also requires us to design experiments to study.

## 5. Conclusion

Our study found that NHE family may regulate the occurrence and development of CRC through tumor immune. In conclusion, we confirmed that NHE family were new diagnostic and therapeutic targets for CRC, which could have important significance for the clinical treatment of CRC.

## Figures and Tables

**Table 1:** The transcription levels of NHE family members in between different types of CRC and colon normal tissues (ONCOMINE).

	Types of colorectal cancer versus normal	Fold change	*t*-test	*p* value	Ref
NHE1	Rectal mucinous adenocarcinoma versus normal Cecum adenocarcinoma versus normal	−2.303 −2.947	−9.275 −11.044	1.29*E*-9 4.96*E*-14	TCGA colorectal TCGA colorectal
	Rectal adenocarcinoma versus normal	−2.951	−15.349	9.66*E*-21	TCGA colorectal
	Rectosigmoid adenocarcinoma versus normal	−2.113	−11.159	3.34*E*-7	TCGA colorectal
	Colon adenocarcinoma versus normal	−3.158	−16.873	8.83*E*-18	TCGA colorectal
	Colon mucinous adenocarcinoma versus normal	−2.638	−8.124	8.44*E*-10	TCGA colorectal
	Rectal adenocarcinoma versus normal	−2.172	−13.622	1.98*E*-26	Gaedcke colorectal
	Colorectal carcinoma versus normal	−2.094	−8.314	9.40*E*-11	Skrzypczak colorectal
	Colorectal carcinoma versus normal	−2.099	−8.476	2.71*E*-9	Hong colorectal
NHE2	Rectal adenocarcinoma versus normal Colorectal carcinoma versus normal Colon carcinoma versus normal Colon carcinoma epithelia versus normal Rectosigmoid adenocarcinoma versus normal Colon adenocarcinoma versus normal Colorectal carcinoma versus normal	−2.492 2.085 −4.579 −4.030 −2.399 −2.209 −6.161	−19.349 −11.349 −13.216 −5.514 −6.146 −8.368 −9.119	6.16*E*-40 5.61*E*-15 3.41*E*-9 5.09*E*-5 1.77*E*-5 7.77*E*-6 2.26*E*-11	Gaedcke colorectal Skrzypczak colorectal Skrzypczak colorectal 2 Skrzypczak colorectal 2 Kaiser colon Kaiser colon Hong colorectal
NHE3	Rectal adenoma versus normal	−3.940	−5.564	1.81*E*-6	Sabates-Bellver colon
	Colon adenoma versus normal	−2.763	−4.237	5.02*E*-5	Sabates-Bellver colon
	Rectal mucinous adenocarcinoma versus normal	−4.816	−6.531	4.37*E*-7	TCGA colorectal
	Rectosigmoid adenocarcinoma versus normal	−5.157	−6.477	1.69*E*-5	TCGA colorectal
NHE4					
NHE5					
NHE6					
NHE7	Colon mucinous adenocarcinoma versus normal	2.169	6.000	3.79*E*-7	TCGA colorectal
NHE8					
NHE9	Rectal adenoma versus normal	−7.304	−8.146	2.69*E*-7	Sabates-Bellver colon
	Colon adenoma versus normal	−2.822	−7.092	2.55*E*-8	Sabates-Bellver colon
	Cecum adenocarcinoma versus normal	−6.006	−12.890	6.32*E*-16	TCGA colorectal
	Rectal adenocarcinoma versus normal	−6.524	−17.291	2.32*E*-23	TCGA colorectal
	Colon mucinous adenocarcinoma versus normal	−4.656	−11.123	5.59*E*-14	TCGA colorectal
	Colon adenocarcinoma versus normal	−6.190	−18.898	8.28*E*-21	TCGA colorectal
	Rectal adenocarcinoma versus normal	−2.659	−16.026	1.27*E*-31	Gaedcke colorectal
NHA2	Cecum adenocarcinoma versus normal	2.392	7.819	6.42*E*-10	TCGA colorectal
	Colon mucinous adenocarcinoma versus normal	2.553	6.438	5.54*E*-8	TCGA colorectal
	Rectal adenocarcinoma versus normal	2.182	9.075	8.87*E*-12	TCGA colorectal
	Colon carcinoma epithelia versus normal	3.418	13.116	6.27*E*-6	Skrzypczak colorectal 2
	Colon carcinoma versus normal	2.544	10.192	2.34*E*-6	Skrzypczak colorectal 2

**Table 2:** GO/KEGG enrichment analysis of NHE family and its 80 interacting proteins.

Category	Term	Description	Count in gene set	*p* value
BP	GO:0038127	ErbB signaling pathway	25	3.88*E*-29
BP	GO:0071900	Regulation of protein serine/threonine kinase activity	31	7.89*E*-24
BP	GO:0018108	Peptidyl-tyrosine phosphorylation	28	1.69*E*-23
BP	GO:0018212	Peptidyl-tyrosine modification	28	1.77*E*-23
BP	GO:0043405	Regulation of MAP kinase activity	25	1.29*E*-21
CC	GO:0045121	Membrane raft	13	3.30*E*-07
CC	GO:0098857	Membrane microdomain	13	3.30*E*-07
CC	GO:0005925	Focal adhesion	13	3.22*E*-06
CC	GO:0030055	Cell-substrate junction	13	3.22*E*-06
MF	GO:1990782	Protein tyrosine kinase binding	12	1.57*E*-12
MF	GO:0019207	Kinase regulator activity	13	1.27*E*-09
MF	GO:0004674	Protein serine/threonine kinase activity	13	1.82*E*-06
MF	GO:0046873	Metal ion transmembrane transporter activity	12	9.48*E*-06
KEGG	hsa04012	ErbB signaling pathway	26	5.28*E*-31
KEGG	hsa05205	Proteoglycans in cancer	33	1.67*E*-30
KEGG	hsa04010	MAPK signaling pathway	30	1.25*E*-21
KEGG	hsa05417	Lipid and atherosclerosis	24	6.06*E*-18
KEGG	hsa05163	Human cytomegalovirus infection	24	1.54*E*-17

## Data Availability

The TCGA data of the gene expression, RNA-seq, and stemness score (RNA-based) of 33 cancers were from the database UCSC Xena. The drug sensitivity processed data were downloaded from the CellMiner™ database.
